# Formative Assessment and Cluster Membership of Homeless Persons’ Emergency Department Visits

**DOI:** 10.7759/cureus.64188

**Published:** 2024-07-09

**Authors:** Gesulla Cavanaugh, Patrick Hardigan, Raymond Ownby, Stachyse Stanis, Prasanna Karur, Raina K Patel, Diti H Patel

**Affiliations:** 1 College of Nursing, Nova Southeastern University, Davie, USA; 2 Health Professions Division, Nova Southeastern University Dr. Kiran C. Patel College of Allopathic Medicine, Davie, USA; 3 Psychiatry and Behavioral Sciences, Nova Southeastern University Dr. Kiran C. Patel College of Osteopathic Medicine, Davie, USA; 4 Medicine, Nova Southeastern University Dr. Kiran C. Patel College of Allopathic Medicine, Davie, USA; 5 College of Allopathic Medicine, Nova Southeastern University Dr. Kiran C. Patel College of Allopathic Medicine, Davie, USA

**Keywords:** readmission risk, substance abuse, cluster analysis, emergency room visits, homeless patients

## Abstract

Background

Evidence is limited on whether homeless individuals who visit emergency departments (EDs) share similar clinical characteristics as individuals from private households who, upon examination, require emergency health services beyond preventative healthcare. While the literature is rich with studies on homeless persons in the ED, a comprehensive assessment of similarities and differences with other social groups is lacking. Consequently, there is a gap in knowledge as it pertains to the appropriate approaches that will further support the development of targeted healthcare and emergency health services for the homeless person.

Aim

The goal is to provide a framework for targeted interventions that hospitals can develop based on behavioral, health, and social characteristics and, in the process, better meet the healthcare needs of homeless patients.

Methods

Data were collected from the records of 85,350 patients in the National Hospital Ambulatory Medical Care Survey (NHAMCS) database who visited the ED between the years of 2013 and 2016; each patient was classified as having either a private residence, living in a nursing home, or homeless (n = 83,446, n = 1,459, and n = 925, respectively).

Results

Cluster analysis of the data confirmed that individuals with multiple comorbidities, as well as substance abuse or dependency, regardless of residence type, were more likely to have recurrent ED visits within 72 hours. Nominal regression analyses revealed that cluster membership generated from ED data could predict patient residence and suggest that substance abuse and depression can predict 72-hour ED visit recurrence.

Conclusion

Cluster analyses have the potential to reveal social health and group characteristics and can support targeted solutions respective to group individualities in the ED.

## Introduction

The chronically homeless person has difficulty accessing the healthcare system appropriately and is inadequately equipped to navigate through available resources for disease management. Habitual alcohol consumption and abuse are often strongly associated with chronic homelessness, unintentional injuries, and life-threatening intoxication. Therefore, it is also a key contributor to the homeless person’s disproportionate use of the emergency department (ED) [1,2], as alcohol abuse can be a risk factor for many chronic illnesses. Mismanagement of chronic disease, when combined with substance abuse, becomes severely detrimental to the homeless person and leads to poor health outcomes. Evidence from the literature asserts that the homeless population has a higher rate of alcohol and substance abuse than housed adults [3,4,5]. Furthermore, because of the homeless person’s constant exposure to relentlessly harsh social and physical conditions, their inability to live a healthy lifestyle is even further hindered.

Although U.S. population estimates point to a steady decline in homelessness nationally from 2007 to 2016, in 2018, there were 552,830 homeless individuals, which was a 0.30% increase from 2017 [6]. Of this number, 358,363 were sheltered, and 194,467 were unsheltered [6]. The U.S. experienced a small increase in homelessness from 2017 to 2018, during which unsheltered homelessness increased by 2% [6]. The U.S. Department of Housing and Urban Development (2018) reported that there was a decrease in overall homelessness for all racial groups except for Caucasians, for whom there was a 4% increase in homelessness overall [7]. Unsheltered homelessness increased among people who identified as White (8% increase), Asian (2% increase), and multiracial (8% increase) [7]. Meanwhile, African Americans were highly represented in the sheltered group, accounting for 47% of the sheltered homeless population and 26% of the unsheltered group [7]. From 2019 to 2020, the homeless population increased by 2.22% [6]. However, from 2020 to 2021, during the first year of the COVID-19 pandemic, a reported 41.59% decrease in homelessness was recorded [6]. Different factors are attributed to this significant decrease, as many reporting resources, such as Point-in-Time counts, were disrupted due to the pandemic. Research within the context of the growth in homelessness and its associated health challenges, which result in significant health disparities, suggests that homeless individuals are one of the most ineffectively treated groups in the ED and elsewhere in the healthcare system. There is evidence to suggest that the significant behavioral health factors mentioned above play a pivotal role in these outcomes [5].

Disproportionate use of the ED is prevalent in the homeless population, particularly in highly populated cities in the U.S. [3,8]. ED visits from individuals experiencing homelessness are often recurrent in a relatively short period. In these cases, individuals may present with a combination of advanced-stage health and social conditions (e.g., depression, intoxication, and hypertension) [3]. Particularly, those over the age of 50 who are homeless and visit the ED are more likely to arrive in critical condition and, subsequently, have a higher risk of mortality [7,8]. Additionally, the multifaceted needs of the homeless person are not fully recognized or addressed in the ED processes [8]. While homeless patients utilize the ED at a higher rate than housed adults, their complex needs for attending the ED may not be met by the protocols for existing services and processes in the hospital [8,9]. Consequently, they are often discharged without receiving targeted health education and the appropriate resources to manage their conditions [8,9].

To examine the characteristics of individuals who visited the ED of selected hospitals in the U.S. at a given time point, this study employed an explorative and machine-learning analytical approach with a particular focus on homeless patients. The aim of the analyses was to assess whether any significant differences existed between individuals who visited the ED based on residence type (private residence, nursing home, or unhoused). Furthermore, because recurrent and preventable ED visits are consistently problematic and can seriously affect hospital operation, we aimed to determine if there were significant differences in the profile of patients who visited the ED more than once within a 72-hour period and whether factors, such as residence type, perception of pain, depression, and ED visit urgency (urgency of the need for an ED visit), could predict the likelihood of ED visit recurrence within 72 hours. The hypotheses for this study are as follows:

Hypothesis 1: There is a significant relationship between the recurrence of ED visits and residence type, perception of pain, depression, and ED visit urgency.

Hypothesis 2: Significant differences in ED patients’ vitals exist based on their characteristics and residence type.

This article was previously posted to the medRxiv preprint server on October 10, 2023.

## Materials and methods

Hospital database

Published hospital data were leveraged from the Ambulatory Health Care Data, made possible by the National Health Care Surveys, which include the National Hospital Ambulatory Medical Care Survey (NHAMCS) [10]. The NHAMCS has been used to assess ambulatory medical care in hospital EDs since 1992. The database contains annual records from approximately 500 nationally representative hospitals within the U.S., where the ED sample data are collected during a four-week reporting window and may vary depending on the hospital. NHAMCS data help yield meaningful descriptive statistics pertaining to the demographics, treatment, medical conditions, diagnostic services rendered, and medication prescribed for those who visit the hospital EDs. The NHAMCS was selected for its robustness in sampling patients’ activities within the ED. For analysis pertaining to this explorative examination, ED visit data were analyzed for 2013-2016 from the NHAMCS database and yielded records on 89,149 patients; patients with unknown residence were omitted from the analyses for a total of 85,830 persons, classified for the purpose of this study as having either a private residence, living in a nursing home, or homeless (n = 83,446, n = 1,459, and n = 925, respectively). Data from previous years before 2013 were excluded mainly due to contrasting naming conventions and variable coding. In 2016, the NHAMCS-ED data were coded based on the International Classification of Diseases, 10th Revision, Clinical Modifications (ICD-10-CM) as opposed to previous years' diagnosis data, which were coded according to the ICD-9-CM. The inclusion criteria for incorporating patient data into the analysis were established based on the following parameters: no missing or invalid entry for 72-hour ER visit recurrence, comorbidity, or residence type. Patients who were younger than 18 years old were excluded from the analyses.

Statistical analysis

To address whether residence type could predict 72-hour ED visit recurrence and the clinical characteristics of the individuals visiting the ED, a series of analyses were performed using JMP v16.0 (JMP Statistical Discovery LLC, Cary, NC) and SPSS Modeler v18.1 (IBM SPSS Statistics for Windows, IBM Corporation, Armonk, NY). Data from 2013 to 2016 were examined, and in-depth analyses were completed on the 2016 database for its inclusion of more study-specific variables, updated ICD-10-CM coding, and for having fewer missing data. The 2016 database contained records on 19,467 patients with more relevant data and variables (i.e., depression and alcohol abuse) relating to the aims of this study; as such, it was selected to address the study hypotheses. Of the 19,467 cases, 650 had unknown residences and were excluded from the analyses.

Additional statistical analyses were conducted to investigate the relationship between residence type, perception of pain, depression, and ED visit urgency. Furthermore, these variables were evaluated to determine whether they were predictive of 72-hour ED visit recurrence. Because the generalized regression procedure fits the generalized linear model (GLM) by penalization to control for overfitting, it was selected for its optimized model estimation capability to assess the most appropriate model for 72-hour ED visit recurrence; a stepwise regression analysis was conducted following the GLM method.

To address the second hypothesis and identify patient grouping characteristics, four-step cluster analyses were applied with different variables. A two-phase process was completed for the analyses involving cluster membership. In the first phase, we used the K-means clustering algorithm to generate three clusters based on the elbow method [11] for ED patients constructed from the selected variables: total comorbidities, 72-hour ED visit recurrence, and substance abuse or dependence. The generated cluster result was saved as a variable assigning a cluster membership for each patient in the database. In the second phase, a nominal logistic regression was applied with cluster membership used as an outcome variable with residence type as a possible predictor.

## Results

Behavioral, health, and social characteristics

In the 2013-2016 dataset, 25% (n = 364) of those living in a nursing home were diagnosed with Alzheimer’s disease (AD) or dementia in comparison to 0.4% (n = 4) in the homeless group and 0.9% (n = 719) in the private household group. Nevertheless, 30% (n = 277) of those classified as homeless reported alcohol misuse, abuse, or dependence, in comparison to 2.9% (n = 2,419) and 1.4% (n = 20) from those in private homes and nursing homes, respectively. Twenty-six percent (n = 240) of those classified as homeless reported having depression, in comparison to 21% (n = 306) of those in nursing homes and 9% (n = 7,510) of those in private households.

**Figure 1:**
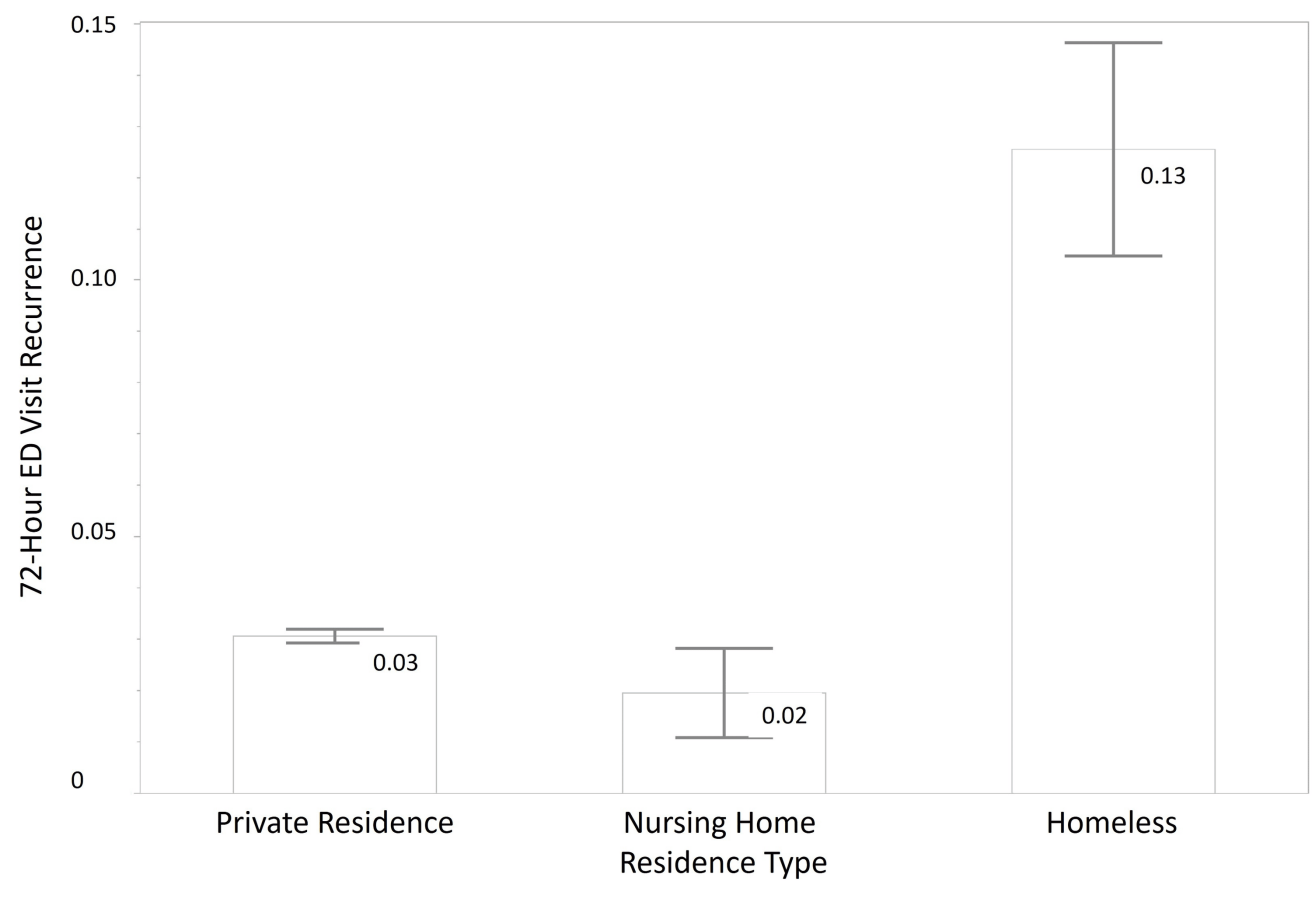
Mean emergency department (ED) visits within 72 hours by residence type ED: emergency department

In the 2016 database composed of 272 patients who were homeless, 287 patients in nursing homes, and 18,258 patients from private residences, further results indicate that patients from the homeless group (M = 0.13, SD = 0.33) were more likely to have recurrent ED visits within 72 hours, compared to those from private homes (M = 0.03, SD = 0.17), and those from nursing homes (M = 0.02, SD = 0.14) (Table 1 and Figure 1). Sixty-nine percent of the homeless group were male. By contrast, in the private residence group, 45% were male, and a similar percentage was observed in the nursing home group (45.30%). Diagnoses of AD/dementia and cardiovascular disease were higher in the nursing home cohort, which is expected as the mean age for that group was higher. The mean age for the private residence group was 37.27 (SD = 23.70), the nursing home group was 73.34 (SD = 18.74), and the homeless group was 43.10 (SD = 14.61). The occurrence of congestive heart failure was 2.7% for the private residence group, 18.5% for the nursing home group, and 1.8% for the homeless group; cerebrovascular disease/history of stroke or transient ischemic attack was 2.8%, 19.2%, and 2.6%, respectively. Following the same trend, the incidence of AD/dementia was highest in the nursing home group at 26.5% and much lower in the housed and homeless groups at 0.9% and 0.7%, respectively. The homeless group had higher diastolic blood pressure mean levels (M = 81.45, SD = 15.12) than either the individuals housed in nursing homes (M = 73.36, SD = 15.71) and those who lived in private residences (M = 78.69, SD = 14.74), but the homeless group had lower systolic blood pressure than both groups (M = 131.70 (19.38), M = 134.54(23.25), M = 137.55(26.21) (Table 1).

**Table 1:** Brief demographic profile by residence type The table displays the means and standard deviations by the three residence types for 72-hour emergency department visit recurrence (72-hour ED), Alzheimer's disease (AD), and systolic blood pressure (BP-Sys).

	Private residence (n = 18,258)		Nursing home (n = 287)		Homeless (n = 272)	
	M(SD)	%	M(SD)	%	M(SD)	%
72-hour ED	0.03(0.17)	-	0.02(0.14)	-	0.13(0.33)	-
AD	-	0.9	-	26.5	-	0.7
BP-Sys	134.54(23.25)	-	137.55(26.21)	-	131.70(19.38)	-

For the 2016 dataset, 5% (n = 921) of the patients in the private residence group reported substance abuse or dependence, compared to 1.5% (n = 5) from the nursing home group and 34.2% (n = 93) from the homeless group. The proportion of patients who reported substance abuse or dependence differed significantly by residence type, *χ*^2^(2, n = 18,817) = 452.19, p < 0.001. Analyses of variance (ANOVA) on residence type yielded significant variation with substance abuse, *F*(2, 18,814) = 214.716, p < 0.001, and with depression, *F*( 2, 18,814) = 62.648. Post hoc Tukey HSD tests showed that for substance abuse, the private residence group did not differ significantly from the nursing home group (p = 0.961) but was significantly different from the homeless group (p < 0.001); the nursing home group was significantly different from the homeless group (p < 0.001). For depression, the private residence group differed significantly from the nursing home group (p < 0.001) and was significantly different from the homeless group (p < 0.001); the nursing home group was not significantly different from the homeless group (p = 0.535). For pain perception, the private residence group significantly differentiated from the nursing home and homeless groups (p < 0.001, p < 0.002), with no difference between the nursing home and homeless groups (p = 0.884). For ED visit urgency, the homeless group differed from the nursing home group (p < 0.042) but not from the private residence group (p < 0.922).

The results from the stepwise regression analysis suggest that with 72-hour ED visit recurrence as the outcome variable, residence type, perception of pain, depression, and ED visit urgency predict a significant amount of criterion variance. The model was significant *F*(4, 17,818) = 5.498, p < 0.0002, R2=.0012. Residence type was the most significant contributor to the model (t = 3.84, p < 0.0001), depression was the second significant contributor (t = 2.10, p < 0.036), while the perception of pain and urgency of ED visit did not contribute significantly to the model (t = 0.56, p = 0.578; t = 1.21, p = 0.221, respectively).

Cluster formation and membership profiles

**Figure 2:**
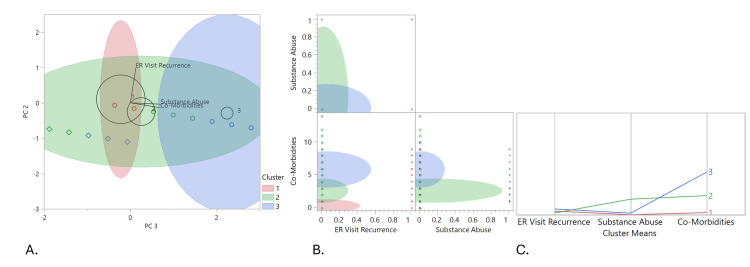
Cluster membership comparison of the three clusters via a (A) biplot, (B) scatterplot matrix, and (C) parallel coordinate plot 1: Cluster 1; 2: Cluster 2; 3: Cluster 3. A: Biplot for two principal components (PCA2 and PCA3). B: Scatterplot matrix for visualization of cluster separation and identification of variable contribution to cluster differentiation. C: Parallel coordinate plot for multidimensional visualization of data; each observation is represented by a line traversing multiple parallel axes corresponding to a specific variable.

Table 2 provides the results of the three-cluster solution generated from three variables selected for their implication in the study aims. The three variables are 72-hour ED visits, substance abuse, and comorbidity. Each cluster is composed of a combination of classifications from the input variables. This clustering process designates the variable with the highest mean in a respective cluster as the identity of that cluster (Table 2 and Figure 2). In this regard, for example, in Cluster 3, comorbidity has a high mean score of 5.844, and as such, that cluster is heavily represented by the comorbidity variable. In the cluster solution, the first cluster (Cluster 1) is mainly composed of patients with no substance abuse or dependency, with low comorbidities, and those who did not have recurrent ED visits in the past 72 hours. The second cluster contains patients who had some comorbidities, with substance abuse and dependency, and with diverse members who did or did not have recurrent ED visits within the past 72 hours. The third cluster is dominated by patients with multiple comorbidities, and some members with substance abuse or dependency and who were more likely to have recurrent ED visits within the past 72 hours, regardless of residence status, than the other two clusters (Figure 2).

**Table 2:** Cluster analysis including comorbidity, substance abuse, and ED visit recurrence This table depicts the three clusters based on 72-hour ED recurrence, substance abuse, and comorbidity. Cluster 1 mainly includes individuals with no substance abuse or dependency, with low comorbidities, and those who did not have recurrent ED visits in the past 72 hours. Cluster 2 contains individuals who have some comorbidities, with substance abuse and dependency, and with diverse members who did or did not have recurrent ED visits within the past 72 hours. Cluster 3 mainly includes individuals who present with multiple comorbidities, substance abuse or dependency, and are likely to have recurrent ED visits within the past 72 hours.

	Cluster 1 (n = 12,727)	Cluster 2 (n = 4,192)	Cluster 3 (n = 782)
	M(SD)	M(SD)	M(SD)
72-hour ED visit	0.035(0.19)	0.017(0.13)	0.056(0.23)
Substance abuse	0.000(0.00)	0.152(0.36)	0.015(0.12)
Comorbidity	0.288(0.46)	2.618(0.86)	5.844(1.29)
Residence type	Count	Count	Count
Private residence	12,208	3,762	690
Nursing home	65	135	55
Homeless	124	118	7

To identify if residence type was associated with cluster membership, a nominal logistic regression was performed using cluster membership as the outcome variable. Analyzing the association between residence type and cluster membership revealed a statistically significant relationship *χ*^2^(4, n = 17,164) = 338.16, p < 0.0001. The model explained 1.4% (R^2^ (U)) of the variance in cluster membership, indicating higher uncertainty accounted for the model; adding other variables of interest, such as depression and diastolic blood pressure, to the model did not significantly improve the proportion of uncertainty (data not shown). Nonetheless, regardless of uncertainty, the cluster analyses included only some variables of interest, which provided a frame of assembly on ED patient characteristics based on residence type.

**Figure 3:**
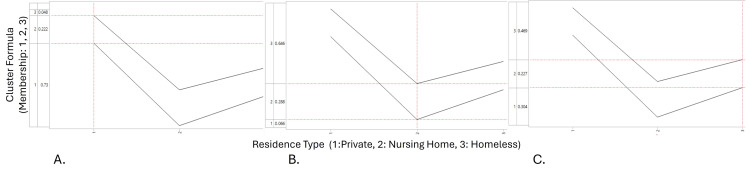
Prediction profiler for cluster membership vs. residence type showing the likelihood by which each residence type is included in each cluster On the vertical line - 1: Cluster 1; 2: Cluster 2; 3: Cluster 3. On the horizontal line - 1: private residence; 2: nursing home; 3: homeless. In the profiler, each residence type is represented by an individual plot (A, B, C), showing the predicted probability of cluster membership on the vertical axis as the predictor values vary on the horizontal axis. The corresponding changes in cluster membership probabilities are observed for (A) private residences, (B) nursing homes, and (C) the homeless.

The model suggests that there is a 0.47 probability that a patient who is homeless will be part of Cluster 3, indicating a higher likelihood for membership in that cluster than Cluster 1 or 2 (0.23 and 0.30, respectively). Recall that Cluster 3 is marked by patients with substance abuse or dependency, multiple comorbidities, and a high likelihood of recurrent ED visits within 72 hours. For patients from nursing homes, there was a 0.65 probability of belonging to Cluster 3. Members of Cluster 1 were more likely to be patients from private residences (0.73), while Cluster 2 represents an equal likelihood of containing patients from any of the three residence types (Figure 3). Following this initial analysis, the prediction profiler was used in JMP to assess likely predictors and determine the robustness of the generated model. In comparing cluster membership with associated covariates, including total chronic illnesses, pulse rate, and age, the results further reveal that Cluster 3 is characterized primarily by older individuals with multiple comorbidities (Figure 3).

## Discussion

The results from this retrospective analysis suggest that there are significantly higher rates of substance abuse, dependency, and depression in individuals who are experiencing homelessness at the time of an ED visit compared to those living in private and nursing homes. Moreover, challenging conditions (a combination of multiple comorbidities and substance abuse) may force individuals from different residences to visit the ED on multiple occasions. The results suggest that it is possible to categorize ED patients based on several factors, including residence type, comorbidities, and depression. Nonetheless, if classifying ED patients with comorbidities or depression alone, it may be challenging to separate the needs of the homeless patient from other groups. Thus, including residence type as a classification category to help provide medical services to patients from the ED may enhance the quality of care of the homeless person. The results are consistent with previous findings, such as Hopkins and Narasimhan (2022), who found that homelessness, coupled with substance abuse and mental illness, aggravates disease burden and early biophysiological deterioration in individuals, which increases the probability of using the ED [12]. Further, identifying and validating homelessness in ED patients can also help with record matching for homeless patients; this allows better identification and standardization of the housing status of ED patients in general. These are crucial as the homeless ED patient presents with unique medical care needs and behaviors [9], which can be captured and addressed with the appropriate evaluation and understanding of the corresponding social determinants of health.

Stricter margins for subsets of individuals with different health and social conditions based on residence type were anticipated for this study; however, using cluster analysis, a distinct cluster membership could not be generated from the dataset that was used in the analysis of homeless patients who visited the ED. This indicates that the homeless persons who present to the ED may have similar health and social characteristics as the other groups, with no significant within-group variance. Moreover, the results suggest, in general, that although the homeless person and the general population have similar health characteristics, the homeless person has a 72-hour ED visit recurrence at higher rates than individuals from private residences. The homeless person may, instead, visit the ED when health issues become unbearable and as a last resort. Future analyses will take within group characteristics into context as applicable to only the homeless person, examining innovative solutions for addressing this group and its presented health challenges.

Limitations

This study was retrospective and used data from 2016, which may not fully represent current ED visit scenarios throughout the U.S. Further, the dataset contained missing data, which could have influenced projected results. Missing data, along with the non-documented medical history of the homeless patients, made it difficult to assess disease burden and overall morbidity.

## Conclusions

The analyses conducted showed that the sampled homeless patients who visit the ED differ in social and medical characteristics from sampled patients from private residences and have a higher rate of 72-hour ED recurrence. Evident indicators are the homeless group’s higher rate of substance abuse but the similarity in depression and pain perception with the nursing home group, which was, on average, 30 years older than the homeless group.

Overall, the results of these analyses suggest that cluster analyses have the potential to reveal social health and group characteristics and can support targeted solutions respective to group individualities in the ED. Potential solutions to address recurrent ED visits from homeless patients include focusing on providing educational information to the homeless patient and encouraging connections to a social worker who may mitigate the circumstances, thereby alleviating worsened health conditions and reducing preventative ED visits.
